# Supplementary material for: UVR8 disrupts stabilisation of PIF5 by COP1 to inhibit plant stem elongation in sunlight
Source: Nat Commun. 2019 Sep 27;10:4417. doi: 10.1038/s41467-019-12369-1 (PMC6764944; doi:10.1038/s41467-019-12369-1)
Supplement: Supplementary file 1 — Supplementary Information [file 41467_2019_12369_MOESM1_ESM.pdf]

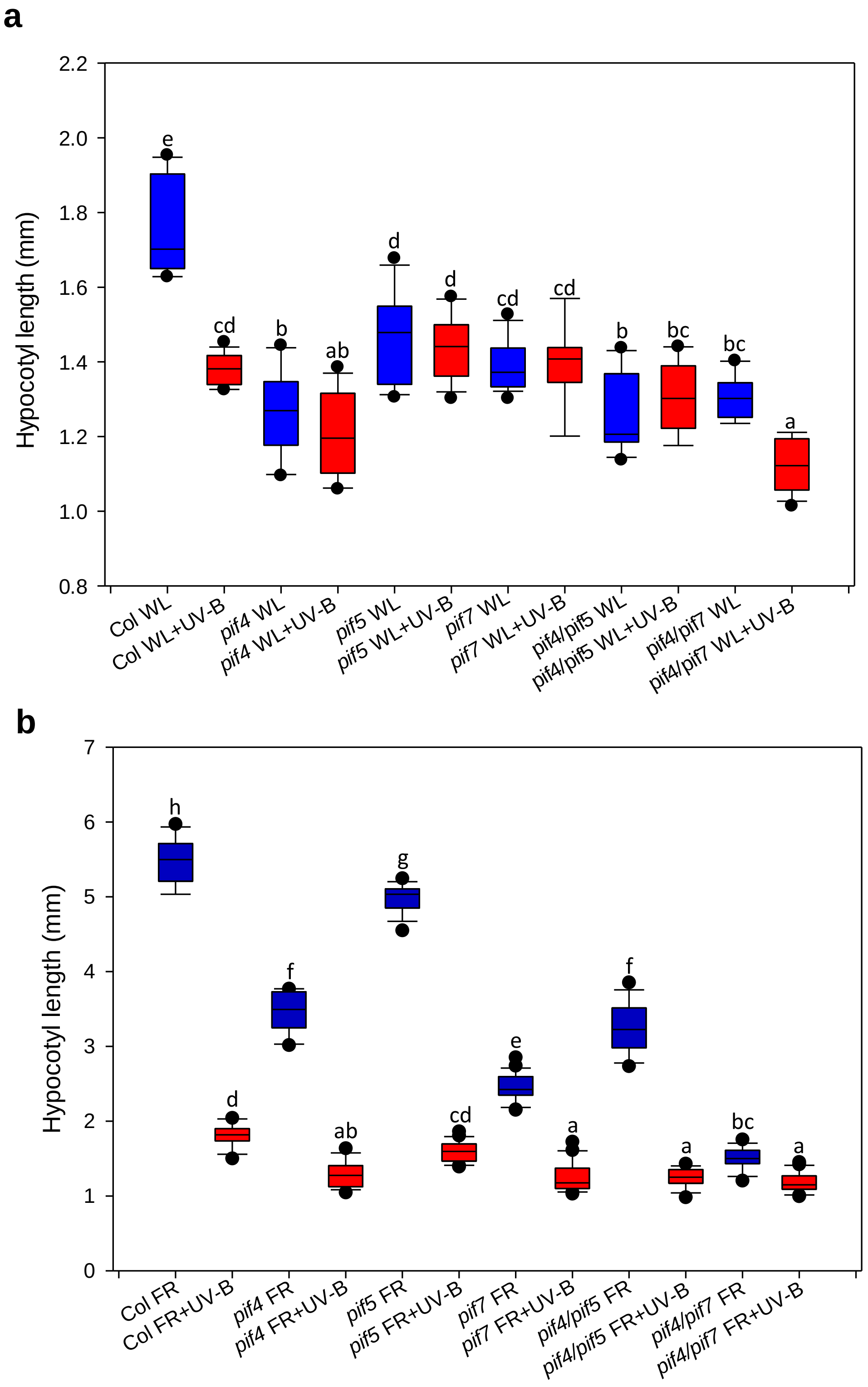

Supplementary Figure 1. UV-B inhibits PIF-mediated hypocotyl elongation.
Hypocotyl length measurements of Col and *pif* mutant seedlings grown for 3 days in continuous high R:FR light before transfer to (**a**) high R:FR (WL) or (**b**) low R:FR (FR) for 4 days ± UV-B. Boxes represent 25th to 75th percentile. Bars show the median and whiskers represent the 10th and 90th percentile outlines. Different letters indicate statistically significant differences between means (Tukey's HSD, $P<0.05$, $n\geq10$). Source data are provided as a source data file.

a

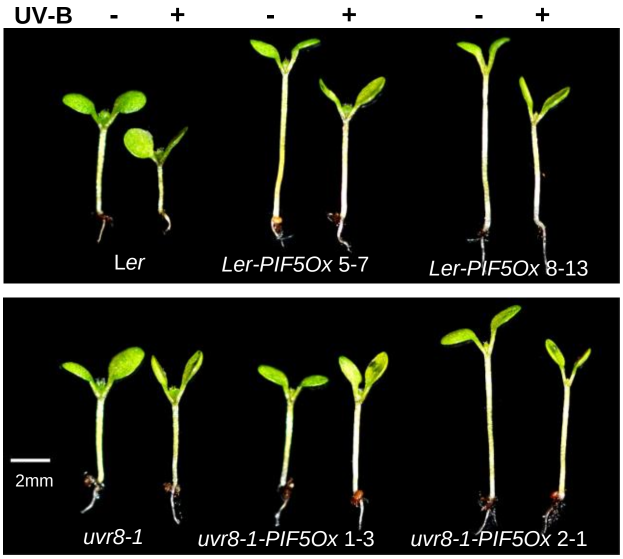

b

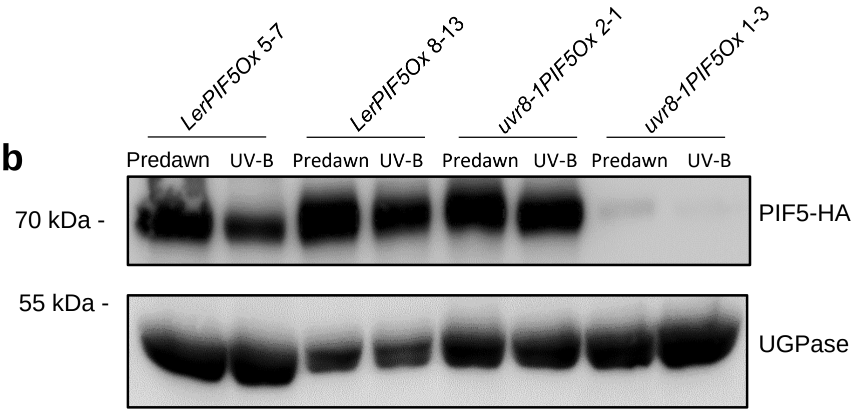

Supplementary Figure 2. Selection of *35S:PIF5-HA* over-expressing lines
a) Two independent *35S:PIF5-HA* lines in L*er* and *uvr8-1* backgrounds were screened for hypocotyl length in high R:FR ± UV-B. Images show representative seedlings of L*er, uvr8-1, LerPIF5Ox* and *uvr8-1PIF5Ox* grown for 3 days in continuous high R:FR before transfer to ± UV-B conditions for 4 days. **b)** PIF5 protein abundance was analysed in *35S:PIF5-HA* lines. Total protein was extracted from 10 day-old seedlings harvested pre-dawn and following a 2 h dawn treatment of white light supplemented with UV-B. Immunoblots were probed with an with anti-HA antibody. UGPase was used as a loading control. *LerPIF5Ox5-7* and *uvr8-1PIF5Ox 2-1* lines showed a similar level of PIF5-HA and were used for further study. Source data are provided as a source data file.

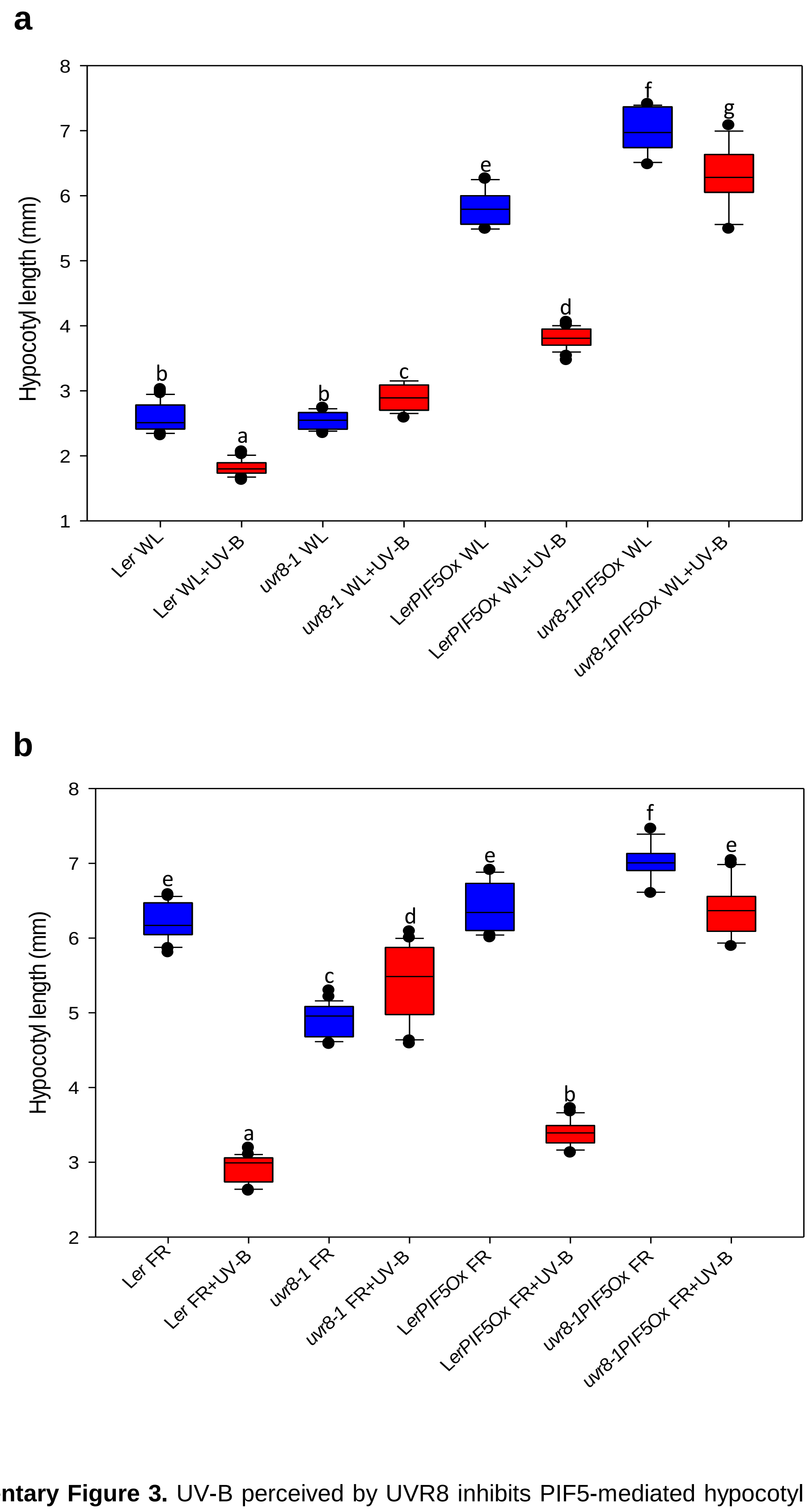

Supplementary Figure 3. UV-B perceived by UVR8 inhibits PIF5-mediated hypocotyl elongation in 16 h light/8 h dark photoperiods.
Hypocotyl length measurements of L*er*, *uvr8-1*, L*erPIF5Ox* and *uvr8-1PIF5Ox* seedlings grown for 3 days before transfer to **(a)** high R:FR (WL) or (**b**) low R:FR (FR) for 4 days ± UV-B. Boxes represent 25th to 75th percentile. Bars show the median and whiskers represent the 10th and 90th percentile outlines. Different letters indicate statistically significant differences between means (Tukey's HSD, $P<0.05$, $n\geq14$). Source data are provided as a source data file.

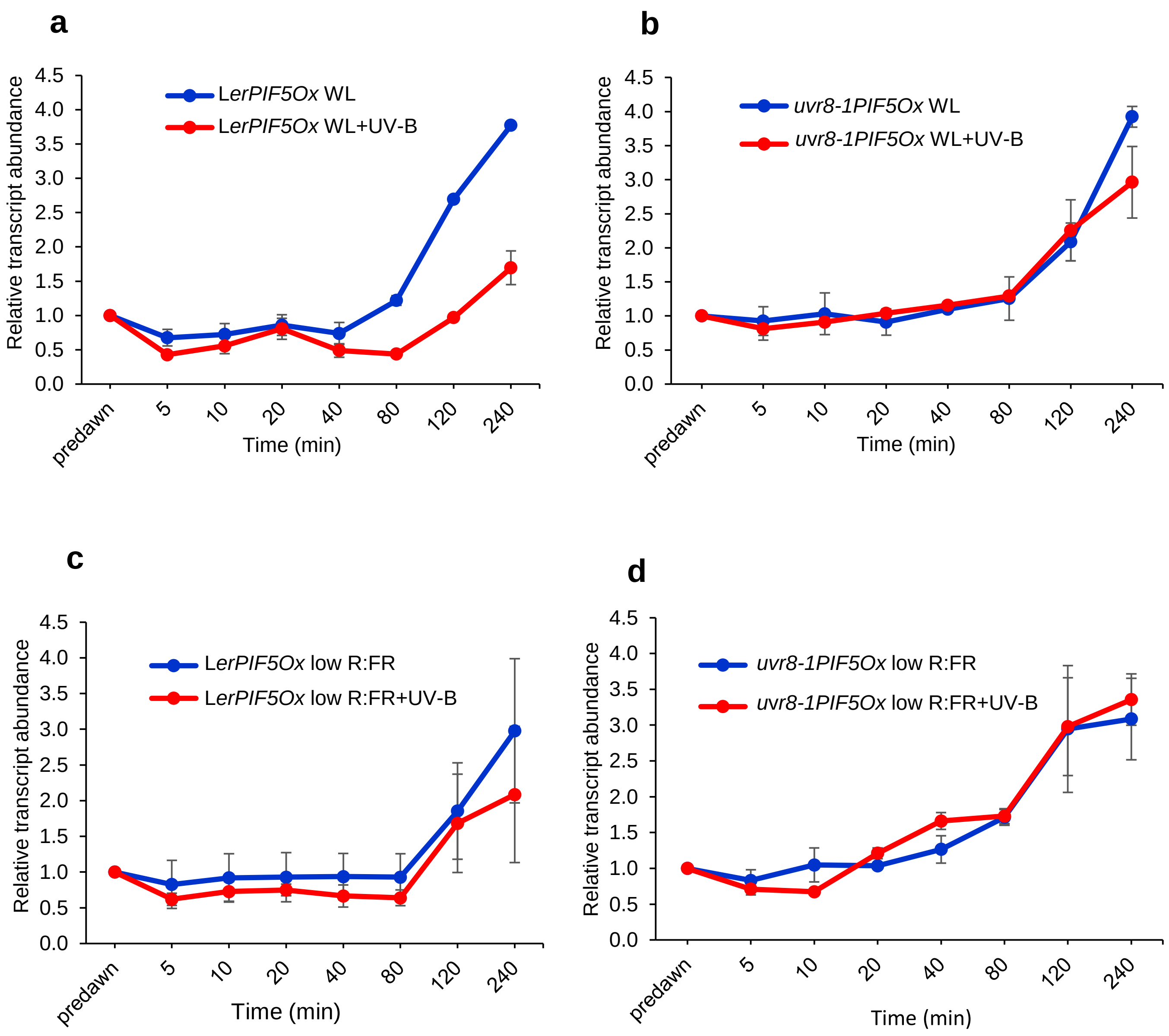

Supplementary Figure 4. UV-B perceived by UVR8 decreases *PIF5* transcript abundance in prolonged high R:FR.

a) Relative *PIF5* transcript in L*erPIF5Ox* and **b)** *uvr8-1PIF5Ox* under high R:FR ± UV-B. **c**) Relative *PIF5* transcript in *LerPIF5Ox* and **d**) *uvr8-1PIF5Ox* under low R:FR ± UV-B. Seedlings were grown for 10 days in 16 h light/ 8 h dark cycles before transfer at dawn to different light conditions. Data represent means of 2 independent biological repeats. Bars represent s.e.m. Source data are provided as a source data file.

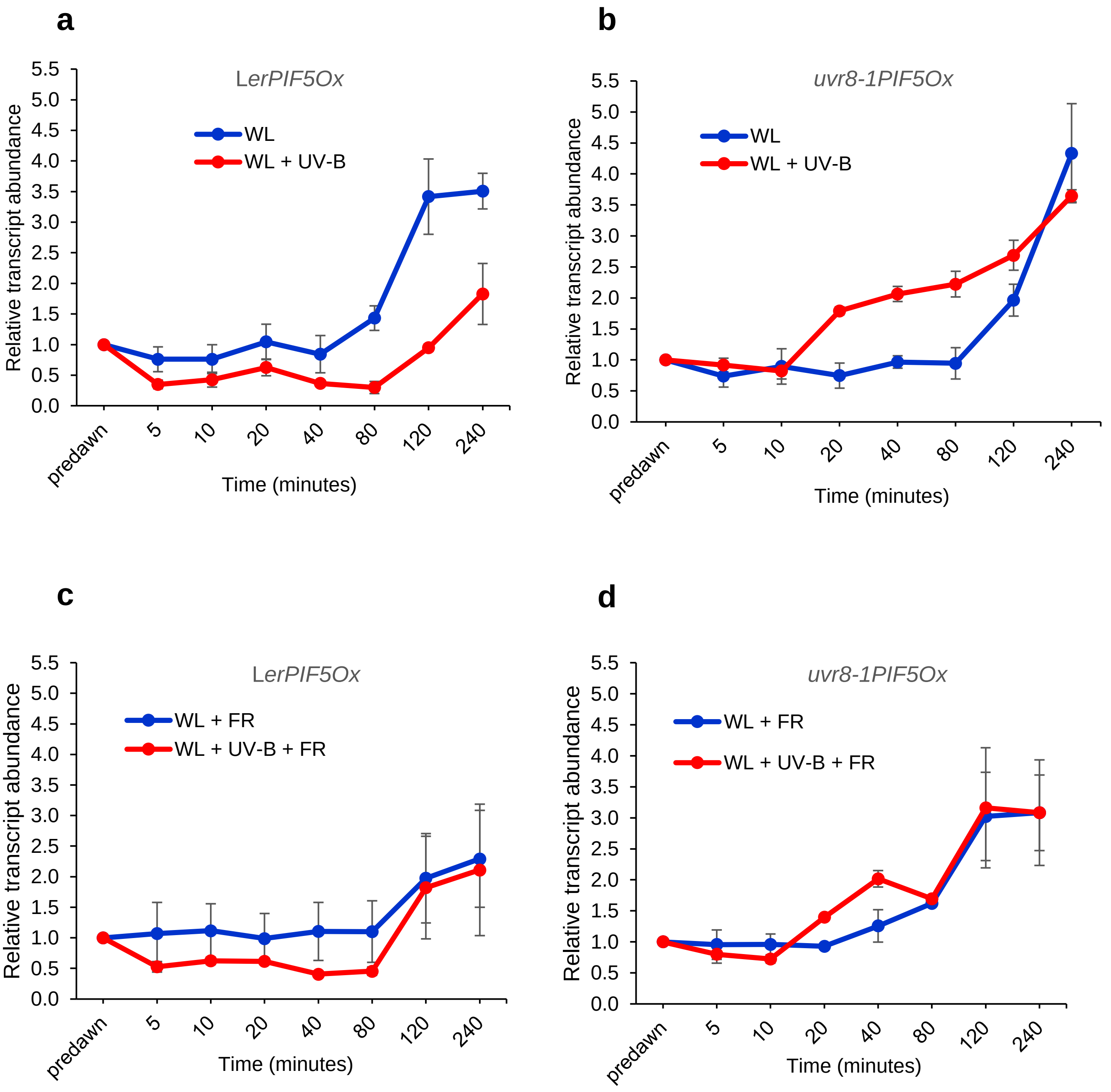

Supplementary Figure 5. UV-B perceived by UVR8 decreases *PIF5-HA* transcript abundance in prolonged high R:FR.

a) Relative PIF5 transcript in *LerPIF5Ox* and b) *uvr8-1PIF5Ox* under high R:FR ± UV-B. c) Relative PIF5 transcript in *LerPIF5Ox* and d) *uvr8-1PIF5Ox* under low R:FR ± UV-B. Seedlings were grown for 10 days in 16 h light/ 8 h dark cycles before transfer at dawn to different light conditions. Data represent means of 2 independent biological repeats. Bars represent s.e.m. Source data are provided as a source data file.

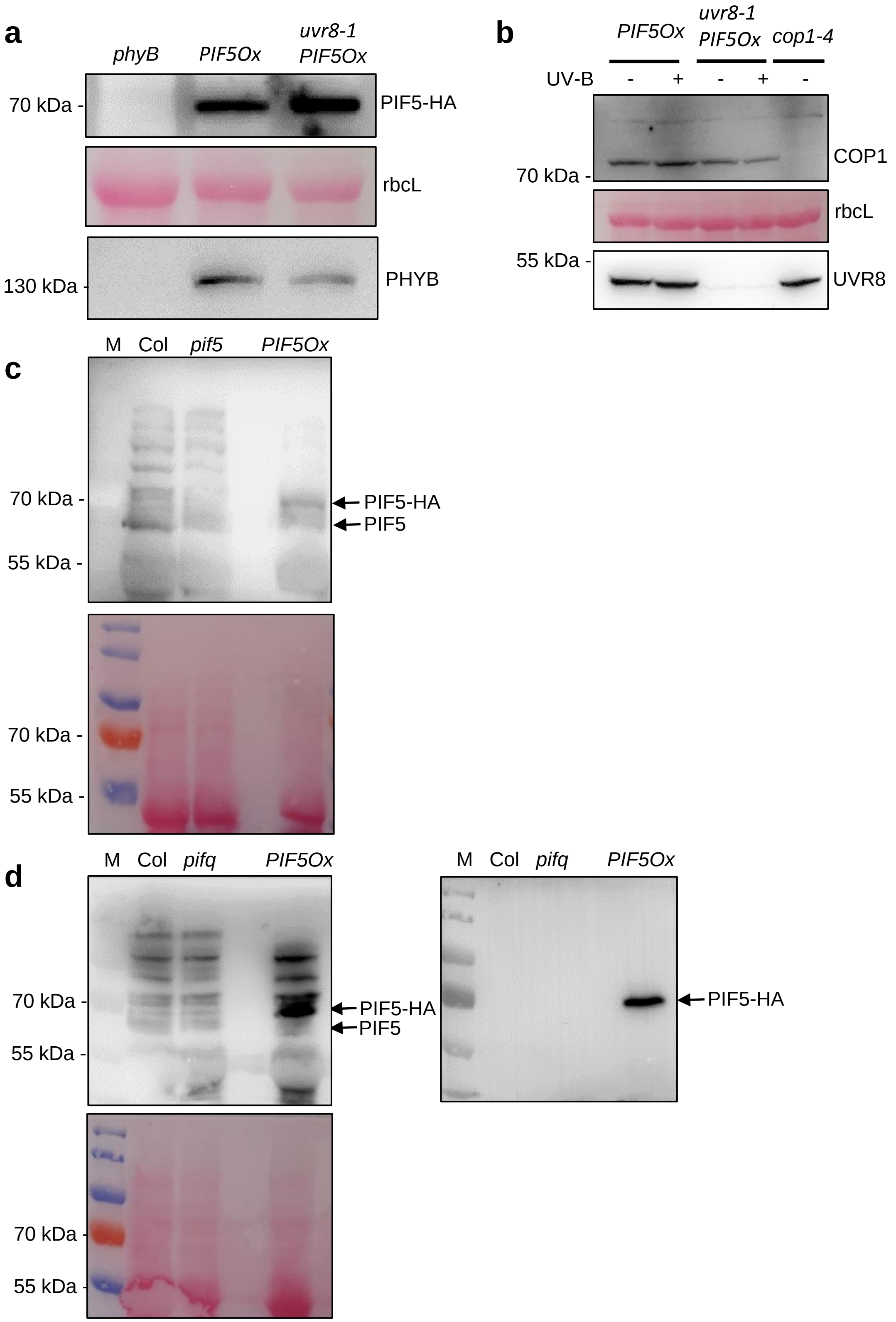

Supplementary Figure 6. COP1, UVR8, PHYB and PIF5 antibody controls

a) *phyB*, *LerPIF5Ox (5-7), uvr8-1PIF5Ox (2-1)* seedlings were grown for 10 days in 16 h light/ 8 h dark cycles before transfer at dawn to WL ± UV-B for 1 h. PIF5 and PHYB were detected with anti-HA and anti-PHYB antibodies, respectively. b) *LerPIF5Ox (5-7), uvr8-1PIF5Ox (2-1)* and *cop1-4* seedlings were grown as in (a). COP1 and UVR8 were detected with aniti-COP1 and anti-UVR8 antibodies, respectively. c) Col, *pif5* and *PIF5Ox* seedlings were grown in the dark for 7 days and harvested at predawn on day 8. PIF5-HA and PIF5 were detected with anti-HA and anti-PIF5 antibodies, respectively. M- marker. Ponceau staining of Rubisco large subunit (rbcL) was used as a loading control. d) Blot produced as in (d) with *pifq* replacing *pif5* (left). This was reprobed with anti-HA antibody (right). Top arrow denotes PIF5-HA and bottom arrow denote PIF5. Source data are provided as a source data file.

a

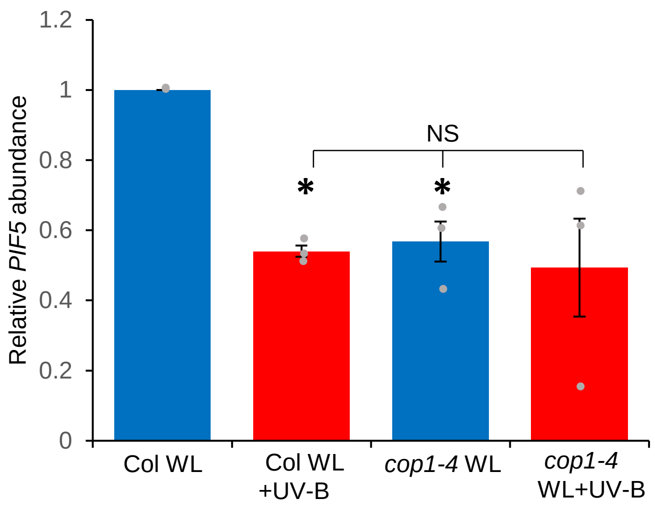

b

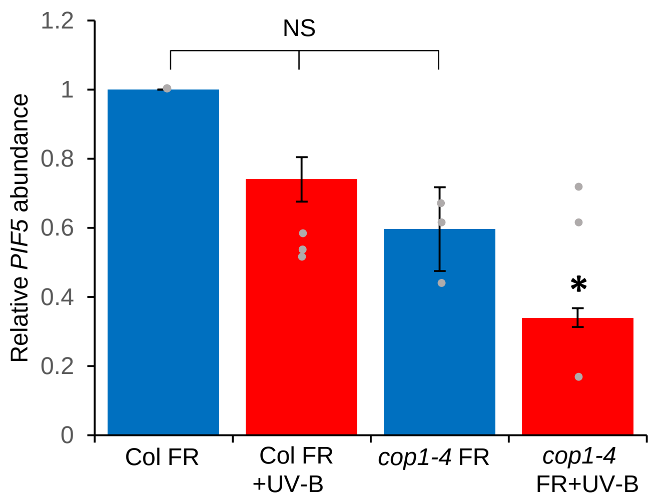

Supplementary Figure 7. COP1 promotes *PIF5* transcript accumulation in high R:FR

Relative *PIF5* transcript abundance in Col and *cop1-4* seedlings grown in (a) high R:FR ± UV-B and b) low R:FR ± UV-B. Seedlings were grown for 10 days in 16 h light/ 8 h dark cycles before transfer at dawn to different light conditions for 2 h. Data represent mean values from 3 biological repeats. Bars represent s.e.m. Student's t-tests were performed on Log2 transformed values ($p<0.05$), ***represents significant decrease in transcript abundance when compared to Col WL (a) or Col FR (b) controls. NS = No significant difference. Source data are provided as a source data file.

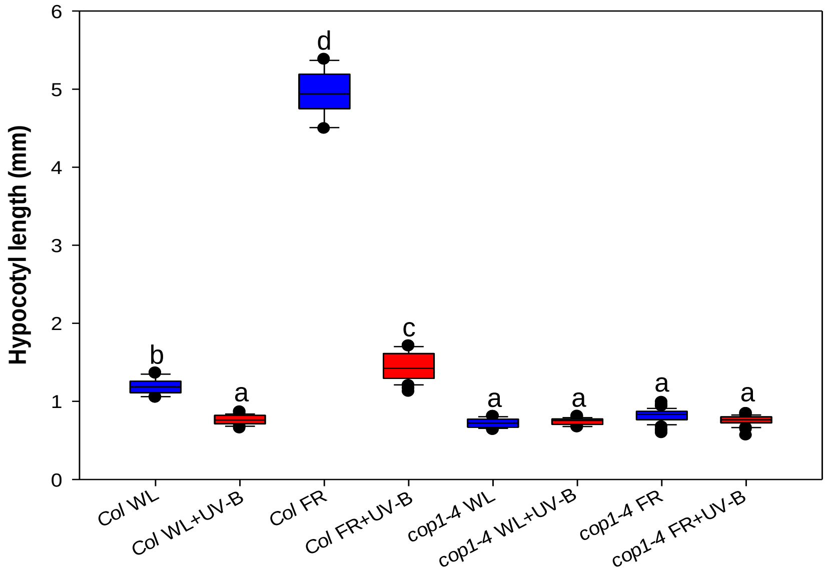

Supplementary Figure 8. *cop1* mutants display no shade avoidance.
Hypocotyl length measurements of Col and *cop1-4* mutant seedlings grown for 3 days in continuous high R:FR light before transfer to high R:FR (WL) or low R:FR (FR) for 4 days ± UV-B. Boxes represent 25th to 75th percentile. Bars show the median and whiskers represent the 10th and 90th percentile outlines. Different letters indicate statistically significant differences between means (Tukey's HSD, $P<0.05$, $n\geq29$). Source data are provided as a source data file.

Primer	Sequence
PIF5 qPCR Forward	CAGATGGCTATGCAAAGTCAGATGC
PIF5 qPCR Reverse	AGATTTGGTTCTGTGCTTGGAGCTG
HA Tag Reverse	GAACGTCGTATGGGTAGTCGACGGAT
ACTIN2 Forward	TCAGATGCCCAGAAGTGTTGTTCC
ACTIN2 Reverse	CCGTACAGATCCTTCCTGATATCC

Supplementary Table 1. List of primers used for qPCR
