# Supplementary material for: UVR8 disrupts stabilisation of PIF5 by COP1 to inhibit plant stem elongation in sunlight
Source: Nat Commun. 2019 Sep 27;10:4417. doi: 10.1038/s41467-019-12369-1 (PMC6764944; doi:10.1038/s41467-019-12369-1)
Supplement: Supplementary file 3 — Reporting Summary [file 41467_2019_12369_MOESM3_ESM.pdf]

nature research

Corresponding author(s): Keara Franklin

Last updated by author(s): 2019/07/15

Reporting Summary

Nature Research wishes to improve the reproducibility of the work that we publish. This form provides structure for consistency and transparency in reporting. For further information on Nature Research policies, see Authors & Referees and the Editorial Policy Checklist .

Statistics

For all statistical analyses, confirm that the following items are present in the figure legend, table legend, main text, or Methods section.

n/a	Confirmed	
☐	☒	The exact sample size (n) for each experimental group/condition, given as a discrete number and unit of measurement
☐	☒	A statement on whether measurements were taken from distinct samples or whether the same sample was measured repeatedly
☐	☒	The statistical test(s) used AND whether they are one- or two-sided<br>*Only common tests should be described solely by name; describe more complex techniques in the Methods section.*
☐	☒	A description of all covariates tested
☐	☒	A description of any assumptions or corrections, such as tests of normality and adjustment for multiple comparisons
☐	☒	A full description of the statistical parameters including central tendency (e.g. means) or other basic estimates (e.g. regression coefficient) AND variation (e.g. standard deviation) or associated estimates of uncertainty (e.g. confidence intervals)
☐	☒	For null hypothesis testing, the test statistic (e.g. F, t, r) with confidence intervals, effect sizes, degrees of freedom and P value noted<br>*Give P values as exact values whenever suitable.*
☒	☐	For Bayesian analysis, information on the choice of priors and Markov chain Monte Carlo settings
☒	☐	For hierarchical and complex designs, identification of the appropriate level for tests and full reporting of outcomes
☒	☐	Estimates of effect sizes (e.g. Cohen's d, Pearson's r), indicating how they were calculated

Our web collection on statistics for biologists contains articles on many of the points above.

Software and code

Policy information about availability of computer code

Data collection	Image J 1.46r was used to obtain hypocotyl length measurements. Evolution CAPT FX6 software was used for protein quantification on western blots. MxPro software was used to quantify relative transcript abundance.
Data analysis	Statistical analysis was performed using IBM SPSS Statistics 24.0 software.

For manuscripts utilizing custom algorithms or software that are central to the research but not yet described in published literature, software must be made available to editors/reviewers. We strongly encourage code deposition in a community repository (e.g. GitHub). See the Nature Research guidelines for submitting code & software for further information.

Data

Policy information about availability of data

All manuscripts must include a data availability statement. This statement should provide the following information, where applicable:

- Accession codes, unique identifiers, or web links for publicly available datasets
- A list of figures that have associated raw data
- A description of any restrictions on data availability

The datasets generated and analysed during the current study are available in the Source Data file. Any other data are available from the corresponding author upon request. There are no restrictions on data availability.

Field-specific reporting

Please select the one below that is the best fit for your research. If you are not sure, read the appropriate sections before making your selection.

☒ Life sciences ☐ Behavioural & social sciences ☐ Ecological, evolutionary & environmental sciences

For a reference copy of the document with all sections, see nature.com/documents/nr-reporting-summary-flat.pdf

Life sciences study design

All studies must disclose on these points even when the disclosure is negative.

Sample size	No sample size calculations were performed. Sample size was selected based on accepted standards in plant molecular biology and determined to be adequate based on consistency and range of the data.
Data exclusions	No data were excluded from this study unless experimental protocols had clearly failed during technique optimization (eg. antibodies did not detect positive controls).
Replication	All experiments were replicated completely independently at least twice. Numbers of biological replicates are stated in figure legends.
Randomization	Plant location in growth chambers was randomised to control for minor variations in light quantity and temperature.
Blinding	Blinding was not possible in hypocotyl assays as experimenters had knowledge of UV-B phenotypes. A logical loading order was required for western blot and co-IP experiments.

Reporting for specific materials, systems and methods

We require information from authors about some types of materials, experimental systems and methods used in many studies. Here, indicate whether each material, system or method listed is relevant to your study. If you are not sure if a list item applies to your research, read the appropriate section before selecting a response.

Materials & experimental systems

n/a	Involved in the study	
☐	☒	Antibodies
☒	☐	Eukaryotic cell lines
☒	☐	Palaeontology
☒	☐	Animals and other organisms
☒	☐	Human research participants
☒	☐	Clinical data

Methods

n/a	Involved in the study	
☒	☐	ChIP-seq
☒	☐	Flow cytometry
☒	☐	MRI-based neuroimaging

Antibodies

Antibodies used	Anti-COP1 antibody was produced by the lab of Ute Hoecker and described in . Balcerowicz et al. (2011) Plant J. 65, 712-723.<br>Anti-PHYB antibody was produced by the lab of Peter Quail and is described in Somers et al (1991) Plant Cell 3, 1263-1274.<br>Anti-HA antibody conjugated to peroxidase was purchased from Roche (12013819001)

Anti-ubiquitin antibody was purchased from Abcam (ab7254)
A polyclonal anti-PIF5 antibody was produced in rabbit by GenScript using the full length PIF5 sequence (AT3G59060) with an N-terminal 6xHis tag

Validation

All antibodies were tested alongside null mutants and (where possible) over-expressing lines to confirm specificity. These blots are provided in the supplementary data.
